# Psychometric validation and measurement invariance of the self-compassion scale-short form (SCS-SF) across gender, clinical population, and cultures

**DOI:** 10.1186/s40359-025-03070-8

**Published:** 2025-07-01

**Authors:** Engin Büyüköksüz, Işıl Tekin, Serkan Arıkan, Şengül İlkay, Atılgan Erözkan

**Affiliations:** 1 Psychological Counseling Center, Istanbul Technical University, Ayazağa Campus Rectorate Building, Maslak, Istanbul, 34469 Turkey; 2 Psychological Counseling and Guidance, Istanbul Medeniyet University, Istanbul, Turkey; 3 Department of Mathematics and Science Education, Bogazici University, Istanbul, Turkey; 4 Department of Psychiatry, Training and Research Hospital, Aksaray University, Aksaray, Turkey; 5 Psychological Counseling and Guidance, Muğla Sıtkı Koçman University, Muğla, Turkey

**Keywords:** Self-compassion, SCS-SF, Validity, Reliability, Measurement invariance, Culture, Türkiye, USA

## Abstract

**Background:**

The concept of self-compassion, rooted in Eastern philosophies, is closely related to psychological well-being and is considered a key skill in alleviating psychological disorders. The short form (SCS-SF) of Self-Compassion Scale was developed and used by many scholars. The current study aimed to conduct an adaptation study of the SCS-SF in Turkish and to analyze its psychometric properties in clinical and non-clinical samples.

**Methods:**

In the adaptation process, the confirmatory factor analysis (CFA) for the general population (*n* = 545) and clinical population (*n* = 246) was conducted. We also investigated evidence for convergent validity (*n* = 274) and conducted a test-retest reliability study with a sample of 53 participants. Additionally, we evaluated the measurement invariance of the SCS-SF across different gender groups, clinical versus non-clinical samples, and cross-cultural samples (comparing Türkiye and the USA, with a sample size of 125 in the USA).

**Results:**

Confirmatory factor analysis showed a six-factor model for the non-clinical sample, yielding fit indices of TLI = 0.920, CFI = 0.953, and RMSEA = 0.095. Also, the six-factor model exhibited a good fit for the clinical sample, with TLI = 0.968, CFI = 0.981, and RMSEA = 0.045. Reliability analysis indicated Cronbach’s alpha values were between 0.77 and 0.88, alongside a strong test-retest reliability coefficient of *r* = 0.878 (*p* < 0.001). Furthermore, measurement invariance was confirmed across clinical and non-clinical groups, as well as across gender groups and among Turkish and American cultural contexts, thereby affirming the scale’s applicability across varied populations.

**Conclusion:**

The adapted SCS-SF scores demonstrated good reliability. The CFA showed a six-dimensional structure and there was evidence for convergent validity and measurement invariance across gender groups as well as between samples from the USA and Türkiye. Consequently, the current study indicates that the adaptation was successful, and the Turkish version can be used for both clinical and non-clinical samples. However, limitations include the reliance on self-report measures and potential cultural bias in the interpretation of self-compassion constructs.

**Supplementary Information:**

The online version contains supplementary material available at 10.1186/s40359-025-03070-8.

## Background

Self-compassion is a concept from Eastern philosophy. Self-compassion could be thought of as a trait (i.e., the tendency of a person to deed or feel compassionate towards oneself within a state or period) and a state (i.e., the level of compassion that an individual shows to oneself in a short period) [1]. Self-compassion is important for the psychological development and well-being of individuals [2]. It is widely acknowledged that self-compassion is a guarding factor contributing to psychological health growth and maintenance [3–5]. Low self-compassion is being studied as a predictor of psychological disorders [6].

Experimental findings revealed that self-compassion is effective in the treatment of certain psychological disorders such as social anxiety, self-harm, borderline personality disorder, suicidal ideation, and eating disorders [7–12]. A meta-analysis of experimental design showed that self-compassion interventions significantly ameliorate different mental disorders (eating behavior, rumination, stress, depression, anxiety) [13, 14]. These studies reported a large effect size for depression symptoms, and eating disorders and a medium effect size for stress, self-criticism, and anxiety as self-compassion is attained [15]. There is still a lack of understanding regarding the correlates of self-compassion, such as social/emotional well-being or mental disorders in adults [16]. That calls for further research in various cultural environments to completely understand these complex relationships. Assessment tools are also needed to collect self-compassion data in multiple languages.

While much of the literature is based on Neff’s six-factor model of self-compassion [1, 3], alternative frameworks also provide valuable insights. One such model is Compassion-Focused Therapy (CFT), developed by Gilbert [17, 18]. CFT conceptualizes compassion as a motivational system rooted in evolutionary and affective neuroscience and emphasizes the interplay between three emotion regulation systems: threat, drive, and soothing. According to CFT, self-compassion is fostered through activating the soothing system, which helps regulate difficult emotions such as shame and self-criticism—particularly relevant in clinical populations [18]. Unlike Neff’s mindfulness- and cognition-based framework, CFT emphasizes the neurophysiological and motivational foundations of compassion and incorporates techniques such as compassionate imagery and self-soothing practices [19]. Integrating these perspectives offers a more comprehensive understanding of the role of self-compassion in emotional regulation and mental health.

## Self-compassion

Self-compassion is acting toward oneself with kindness, mindfulness, and common humanity against challenges in life and personal incompetence [20]. If an individual fails to show compassion to oneself, that individual will end up in a self-judgment, isolation, and over-identification. Self-compassion is the conceptualization of the process in which the individual reacts emotionally toward pain (with more kindness and less criticism), cognitively understands pain (as a part of the human experience rather than isolation), and experiences differences between these states (with more awareness and less identification) [21]. Neff [20, 22] defined six factors of self-compassion: self-kindness, common humanity, mindfulness, self-judgment, isolation, and over-identification.

Self-kindness is the individual’s ability to be kind to one’s pain and respond to the request to alleviate this pain. Common humanity includes naming the joint experience of human flaws and understanding that all humans may fail and make mistakes. Mindfulness is the awareness of the existing pain experience with calmness and balance. Self-compassion will help individuals act kindly toward personal weaknesses or challenging situations in life by understanding that this is human nature and by raising awareness of custom experiences (personal emotions and thoughts) [20]. Self-judgment is the strict criticism of self because of personal failure. Isolation involves feeling lonely during a painful experience. Over-identification emerges when individual thoughts merge which leads to the loss of perspective due to pain. Self-compassion against self-judgment is human nature against isolation or awareness against over-identification. The six factors of self-compassion mutually affect each other and it is believed that these factors interact as a system [23].

### The self-compassion scale (SCS) and the self-compassion scale-short form (SCS-SF)

Neff [20] proposed and demonstrated that the SCS is a measure of self-compassion based on six-factorial structure [22]. Neff and colleagues [24] conducted a confirmatory factor analysis (CFA) study that evaluated the six-factorial structure and found that a single high-order self-compassion structure fit the data marginally well. Other studies also have found that the SCS has a six-factorial structure in Spain [25], in Portuguese [26], a two-factor structure in Italian [27] and Portuguese [28], and a single-factor structure in Turkish [29]. For the French adaptation of the scale, Kotsou and Leys [30] revealed the bifactor solution while for the Chinese language adaptation, Zeng and colleagues [31] proposed a three-factor model for the positive items and a one-factor model combining the negative items. For the Hungarian language, Tóth-Király and colleagues [32] verified the practicality of the bifactor ESEM structure.

Raes and colleagues [33] shortened the SCS and created a 12-item and six-factor short form in both Dutch and English. The Self-Compassion Scale-Short Form (SCS-SF) was produced by selecting the two highest correlating items from each of the six factors of SCS [33]. The SCS-SF has exhibited robust internal consistency and reliability across various samples, including individuals with dementia, elderly populations, and university students [34–36]. This has contributed to its widespread use in measuring self-compassion among both clinical and non-clinical groups [26, 34, 35]. However, language adaptations of the SCS-SF have revealed that the factorial structure of the SCS-SF varies among cultures. For example, Spain [25] has six-factor structures, Canada [37], Spain [38], Sweden [34], the United Kingdom [35, 36], and the United States [39] have two-factor models, while the Chinese [40] have three-factor models. This divergence underscores the intricate nature of the construct and prompts inquiries into the scale’s suitability across various cultural and clinical environments. Cultural differences have significantly shaped the factorial structure of the SCS-SF assessments. Spanish samples demonstrated the viability of both six-factor models [25, 33] and the two-factor model, being particularly effective [34–39]. In contrast, among Chinese nursing students and healthcare professionals, a three-factor model was identified, distinguishing one positive dimension from two negative ones, thereby underscoring the influence of cultural and linguistic variances on the interpretation of the scale [40]. These results have demonstrated the considerable applicability of the SCS-SF, but the factor structure of the SCS-SF is an issue that needs to be examined in terms of cross-cultural sensitivity.

### Measurement invariance

Gender studies, as evidenced by the work of Neff and colleagues [24], indicate that both men and women have a comparable understanding of the Self-Compassion Scale-Short Form (SCS-SF), which facilitates valid comparisons between genders. Meta-analytic research has consistently demonstrated that males tend to exhibit marginally higher levels of self-compassion compared to females, with small effect sizes [41–43].

Investigations within clinical populations, including studies by Costa and colleagues [28] and Neff and colleagues [44], affirm the SCS-SF’s relevance in clinical contexts, preserving its structural integrity even among individuals prone to heightened self-criticism, such as those experiencing anxiety or depression. Furthermore, cross-cultural validation efforts, as highlighted by Neff and colleagues [24] and Tóth-Király and colleagues [32], demonstrate the SCS-SF’s comparability across various languages and cultural backgrounds. Nonetheless, it is important to note that cultural norms may affect the visibility of certain subcomponents, such as self-kindness about mindfulness. In summary, the SCS-SF is a flexible instrument, supported by substantial evidence for its applicability across different groups and its capacity to provide nuanced insights into specific populations.

### Present study

Since the SCS-SF is commonly used in various cultures to assess self-compassion, its availability in Turkish is anticipated to facilitate its application in research including Turkish participants. However, to date, no validated short-form Turkish version of the SCS-SF exists, which limits the ability to conduct cross-cultural comparisons and hinders its use in Turkish-speaking clinical and non-clinical populations. This gap in the literature necessitates an adaptation study that ensures both linguistic and psychometric validity. Thus, the following psychometric evaluations were conducted: (i) the factor structure of the SCS-SF, with an emphasis on potential samples differences; (ii) internal consistency/reliability and test-retest of the SCS-SF in clinical and non-clinical samples; (iii) concurrent validity of the SCS-SF, hypothesizing negative correlation with depression, anxiety, and Rosenberg Self-Esteem Scale whereas positive correlation with the positive relations with others subscale of psychological well-being; (iv) measurement invariance by gender, clinical samples (clinical vs. non-clinical), and cultural samples (Turkish vs. American). The SCS is frequently employed to evaluate the results of both meditation-focused and clinical interventions [45, 46], which underscores the significance of incorporating these populations into our study. Since different factorial structures have been found in cross-cultural studies of the SCS-SF, it was thought that comparison with the original sample of the scale (American) would support the hypotheses of the adaptation study.

## Method

### Study design

An observational cross-sectional design was used, appropriate for the study’s aim of validating the Turkish SCS-SF. This design allows for the examination of psychometric properties across diverse groups in real-world conditions, which is essential in scale adaptation research.

### Participants

The current study included a total of 1243 participants to provide reliability and validity evidence of SCS-SF scale adaptation (Table 1). For factor structure, internal consistency and measurement invariance, 545 non-clinical participants (206 male and 339 female undergraduate students in a Turkish university, *M*_age_ = 22.64) and 246 clinical participants (84 male and 162 female, *M*_age_ = 29.51) were included. Clinical participants, living in Türkiye, were diagnosed with depressive disorders according to DSM-V criteria. Test-retest reliability was analyzed with 53 Turkish non-clinical undergraduate students (11 male and 42 female, M_age_ = 20.64). To provide convergent validity evidence of SCS-SF, 274 Turkish non-clinical undergraduate students (82 male and 192 female, M_age_ = 21.04) were included in the study.

**Table 1:** Descriptive information of the participants

	Non-clinical sample Confirmatory Factor Analyses	Clinical sample Confirmatory Factor Analyses	Test-Retest	Correlations with other scales for convergent validity	American non-clinical participant
*n*	545	246	53	274	125
Average age (range)	22.64 (18–45)	29.51 (18–62)	20.64 (18–26)	21.04 (18–43)	34.60 (18–68)
% female	62.2% (*n* = 339)	65.9% (*n* = 162)	79.2% (*n* = 42)	70.1% (*n* = 192	70.4% (*n* = 88)
% male	37.8% (*n* = 206)	34.1% (*n* = 84)	20.8% (*n* = 11)	29.9% (*n* = 82)	28.0% (*n* = 35)
% other	-	-	-	-	1.6% (*n* = 2)
Internal consistency (Cr𝝰)	0.81	0.77		0.81	0.88

To evaluate the measurement invariance across Turkish and American samples, data were collected in the United States by sharing it via the mailing list of the Association for Contextual Sciences. The American sample consisted of 125 non-clinical participants (35 male and 88 female, M_age_ = 34.60). Since the aim was not to make a direct comparison and the researchers encountered difficulty finding volunteer participants living in the USA, a small but still sufficient American subsample was used. Hair and colleagues [47] and Kline [48] (2011) stated that 10 participants per item would be sufficient for factor analysis and Wolf and colleagues [49] stated that small sample groups are sufficient for simple factor structures [47–49]. Sample size was determined based on established guidelines suggesting a minimum of 10 participants per item for confirmatory factor analysis (CFA) (47,48). With 12 items in the SCS-SF, the American subsample of 125 was considered statistically adequate. While a formal a priori power analysis was not conducted, recent simulation studies suggest that adequate model estimation is possible in simple CFA models even with smaller samples, especially when factor loadings are moderate to high and models are properly specified (49). The demographic breakdown of the American non-clinical participants included 79 White, 20 Latino, 12 Asian American, 9 European, 8 Hispanic, and 5 Afro-American.

The ethical committee of the first author’s university gave its approval to the current study (protocol no: 180197). A written informed consent form was given to all the participants and participants gave their consent to be included in the study. All the data were collected face-to-face, except for the American participants.

### Instruments

#### Self-compassion scale-short form (SCS-SF)

The SCS-SF is the short form of 26-item SCS developed by Neff [22]. Raes and colleagues [33] shortened the SCS scale and created a 12-item and six-factor form. The responses of SCS-SF are given on a 5-point Likert scale (1 = almost never to 5 = almost always). The current study adapted SCS-SF into Turkish and the sample items of the English and Turkish forms are presented in Table 2.

**Table 2:** Sample items of the SCS-SF

Subscale	Sample Item in English	Sample Item in Turkish
Self-Kindness	2. I try to be understanding and patient towards those aspects of my personality I don’t like.	Kişiliğimin hoşlanmadığım yönlerine karşı anlayışlı ve hoşgörülü olmaya çalışırım.
Self-Judgment	11. I’m disapproving and judgmental about my own flaws and inadequacies.	Sahip olduğum kusurlarımı, yetersizliklerimi onaylamıyorum ve yargılıyorum.
Common Humanity	5. I try to see my failings as part of the human condition.	Başarısızlıklarımı insan doğasının bir parçası olarak görmeye çalışırım.
Isolation	4. When I’m feeling down, I tend to feel like most other people are probably happier than I am.	Kendimi üzgün hissettiğim zaman, çoğu insanın muhtemelen benden daha mutlu olduğunu hissetmeye meyilli olurum
Mindfulness	3. When something painful happens I try to take a balanced view of the situation.	Acı verici bir şey olduğu zaman, durumu dengeleyen bir bakış acısı almaya çalışırım.
Over-identified	1. When I fail at something important to me I become consumed by feelings of inadequacy.	Benim için önemli olan bir şeyde başarısız olduğumda, kendimi yetersizlik duygusuyla tüketirim

#### Convergent validity evidence

In order to provide convergent validity evidence for SCS-SF adaptation, Rosenberg Self-Esteem Scale, scales of Psychological Well-being, Beck Depression Inventory and The State-Trait Anxiety Inventory were used. A brief description of each scale is provided below.

#### Rosenberg self-esteem scale (RSES)

The RSES [50] was utilized to provide convergent validity evidence for the SCS-SF for non-clinical sample. The RSES consists of five positive and five negative items assessing self-esteem. The items are rated on a 4-point Guttman scale (0 = completely disagree; 3 = completely agree). As the RSES is a Guttman scale, the scoring of the RSES involves a method of combined ratings [51]. The total score received from the scale ranges from 0 to 6, with high scores indicating low self-esteem. Cuhadaroglu [52] reported that the test-retest reliability of the Turkish version of the RSES was 0.75.

#### Scales of psychological well-being (SPWB)

The “positive relations with others” subscale of the SPWB [53] was utilized to provide convergent validity evidence of the SCS-SF for non-clinical sample. As the research indicated [54, 55], a moderate positive correlation between the SCS-SF and the SPWB’s “positive relationships with others” subscale is anticipated. The items of SPWB are rated on a 6-point Likert scale (1 = strongly disagree; 6 = strongly agree). The Turkish version of the “positive relations with others” subscale has Cronbach’s alpha of 0.89 [56]. In the current study, the internal consistency coefficient of “positive relations with others” was calculated as 0.85.

#### Beck depression inventory (BDI)

The BDI was utilized to provide convergent validity evidence of the SCS-SF for the clinical sample. A moderate negative correlation between the SCS-SF and the BDI was anticipated, as other studies indicated the SCS-SF to be associated with depression in clinically depressed persons [57]. The items of BDI are rated on a 4-point Likert scale (0–3). A total score ranging from 0 to 63 can be obtained from BDI. The Cronbach’s alpha value and split-half reliability coefficient of the Turkish version of the BDI were reported as 0.80 and 0.74, respectively [58–60]. According to the current study, Cronbach’s alpha of BDI in the clinical sample was estimated as 0.91.

#### The state-trait anxiety inventory (STAI)

STAI was developed by Spielberger [61], 20 items measuring state anxiety (STAI-S) and 20 items measuring trait anxiety (STAI-T). The STAI-S was utilized to provide convergent validity evidence of the SCS-SF in a clinical sample. Items are scored on a 4-point Likert scale (1 = not at all; 4 = very much so). The total score received from the scale ranges from 20 to 80, with higher scores indicating a higher level of anxiety. According to the Turkish adaptation study, Cronbach’s alpha value for the STAI-S was 0.76 [62]. A moderate and negative correlation between the SCS-SF and the STAI-S was anticipated due to prior studies demonstrating a similar relationship in clinical settings [63]. According to the current study, Cronbach’s alpha of state anxiety in the clinical sample was estimated as 0.88.

#### Procedure of SCS-SF adaptation

For the adaptation of SCS-SF into Turkish, the processes outlined in the International Test Commission (ITC) guideline were followed [64]. Permission has been sought and approved for the Turkish adaptation of the SCS-SF (Kristin Neff, personal contact, April 23, 2018). Depending on the ITC Guidelines for Translating and Adapting Tests we used the multiple translation design. Three experts independently translated the scale into Turkish. The experts were native Turkish researchers with advanced English skills who were familiar with the scale development and general testing principles. Following that, an expert panel was convened to reconcile with these experts. The draft Turkish form was reviewed for semantic, cultural, and grammatical aspects, as well as for accuracy. The experts discussed each item and agreed on the items. For instance, the experts decided to use “when I feel sad” instead of “when I feel bad”, “the self-attention and self-compassion I need” instead of “the attention and compassion I need”, and “my dislikes about my personality” instead of “my dislikes about myself”. Then, a backward translation was conducted by an independent translator. The comparison of the original and retranslated versions revealed that the Turkish and original versions of the scale had no semantic differences.

### Data analysis

The data analysis part included descriptions of how to evaluate the factor structure of SCS-SF, reliability and validity evidence of scores as well as how to verify measurement invariance across groups. In the current study, missing data were handled using listwise deletion. The data analysis included a range of statistical techniques: descriptive statistics (means, standard deviations), reliability analyses (Cronbach’s alpha, test–retest correlations), Pearson correlation coefficients for convergent validity, confirmatory factor analysis (CFA) using the WLSMV estimator, and multi-group CFA for measurement invariance testing (configural, metric, scalar). Additionally, bias-corrected and accelerated (BCa) 95% confidence intervals were computed for correlation estimates using bootstrap resampling (1,000 iterations). Model fit was evaluated using χ², CFI, TLI, RMSEA, and WRMR fit indices.

### Testing the factor structure

The factor structure of the SCS-SF was tested with confirmatory factor analysis, using the weighted least squares means and variance adjusted (WLSMV) estimation method. One-factor, two-factor, six-factor, two-bifactor, and six-bifactor models were examined in order to obtain accurate interpretations as the literature has divergent findings on the number of SCS-SF factors. In the two-bifactor model, one general factor and two uncorrelated factors were used whereas in the six-bifactor model one general factor and six uncorrelated factors were used. The factorial structure of the SCS-SF was assessed for clinical and non-clinical. Chi-square test statistics, the Tucker‒Lewis index (TLI), comparative fit index (CFI), root mean square error of approximation (RMSEA), and weighted root mean square residual (WRMR) fit indices were used to estimate the model fit to the data [65–68]. To determine the best possible model, different fit indices are also utilized because chi-square is sensitive to large sample sizes and may produce inflated Type-1 error. TLI and CFI values higher than 0.95 [48], RMSEA value less than 0.06 [69], and WRMR value less than 0.90 represent good fit [70]. IBM SPSS 22.0 and Mplus 7.4 [71]. statistical package programs were used to analyze the data.

### Reliability

To evaluate the internal consistency, Cronbach’s Alpha value was reported. If the internal consistency value is less than 0.70, internal consistency is problematic; 0.70 to 0.80 is acceptable; 0.80 to 0.90 is good; and above 0.90 is excellent [72]. The test-retest method [73] and internal consistency evaluation were used to assess the reliability of SCS-SF scores. For test-retest reliability, 53 participants took the SCS-SF twice over a two-week period, and the Pearson correlation between these two implementations was reported.

### Convergent validity

Convergent validity of the SCS-SF was assessed for the clinical sample using the BDI and the STAI-S, and for the non-clinical group using the RSES and the “positive relations with others” subscale of the SPWB. Based on previous research findings and theoretical relationships [22, 23, 44, 74, 75], for the non-clinical sample, a positive correlation between SCS-SF and the SPWB (positive relations with others subscale) was expected, whereas a negative correlation between SCS-SF and the RSES were expected. Furthermore, for the clinical sample, negative correlations between the SCS-SF and the BDI and the STAI-S were expected. Pearson correlation coefficients (*r*) were reported. In accordance with current best practices, bias-corrected and accelerated (BCa) 95% confidence intervals were computed for all correlation estimates using bootstrapping (with 1,000 resamples) to provide more robust estimates of precision and to account for potential non-normality in the data distributions [48].

### Measurement invariance (MI)

Using the multi-group CFA, the measurement invariance of the SCS-SF was examined across gender groups, cross-cultural groups (Turkish versus American), and clinical versus non-clinical groups. To this end, configural, metric, scalar, and strict invariance [76] were examined hierarchically. First, the equality of the factorial structure of the SCS-SF was estimated (configural invariance). Then, the equality of factor loadings was examined (metric invariance). Then, to test the scalar invariance, thresholds were constrained to be the same across groups. Finally, the equality of error variances was examined (strict invariance). The ΔCFI and ΔRMSEA values are expected to be lower than 0.010 and 0.015, respectively [77].

## Results

### Testing factor structure

CFA was used to evaluate the factorial structure of the SCS-SF for clinical and non-clinical samples. To decide the best fitting model in the Turkish sample, one-, two-, six-factor, and two-bifactor and six-bifactor models were compared.

### Factor structure for non-clinical sample

**Fig. 1:**
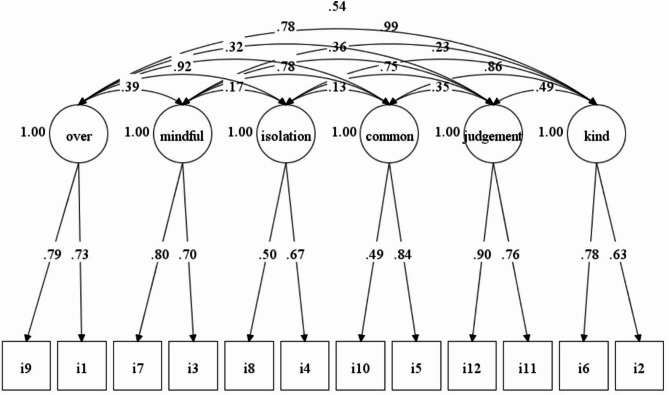
Standardized factor loadings of the items for non-clinical sample

As presented in Table 3, for a non-clinical Turkish sample, the CFA outcomes showed that the fit of the six-factor model and two-bifactor model was similarly good (TLI = 0.920, CFI = 0.953, RMSEA = 0.095, WRMR = 1.115 for the six-factor model; TLI = 0.931, CFI = 0.956, RMSEA = 0.088, WRMR = 1.000 for the two-bifactor model). Thus, the factor structure proposed by Raes and colleagues [33] was supported by a non-clinical Turkish sample. Furthermore, the standardized factor loadings ranged between 0.49 and 0.90 (Fig. 1), indicating that the relationships between items and factors were in the expected direction and magnitude.

**Table 3:** CFA results of the SCS-SF

Sample	Models	X^2^	df	X^2^/df	TLI	CFI	RMSEA (%90 CI)	WRMR
Non-clinical sample	One-factor	1304.467	54	24.156	0.621	0.690	0.206 (0.197–0.216)	3.353
	Two-factor	377.003	53	7.113	0.900	0.920	0.106 (0.096–0.116)	1.541
	Six-factor	230.517	39	5.910	0.920	0.953	0.095 (0.083–0.107)	1.115
	Two-bifactor	219.242	42	5.220	0.931	0.956	0.088 (0.077-0.100)	1.000
	Six-bifactor	Not identified						
Clinical sample	One-factor	263.459	54	4.882	0.753	0.798	0.13 (0.11–0.14)	1.364
	Two-factor	116.649	53	2.200	0.924	0.939	0.070 (0.053–0.087)	0.874
	Six-factor	58.611	39	1.502	0.968	0.981	0.045 (0.017–0.068)	0.586
	Two-bifactor	88.168	42	2.099	0.930	0.956	0.067 (0.047–0.086)	0.694
	Six-bifactor	Not identified						

Note. SCS-SF = Self-Compassion Scale Short Form; TLI = Tucker–Lewis index; CFI = comparative fit index; RMSEA = root mean square error of approximation; CI = confidence interval; WRMR: Weighted Root Mean Square residual

### Factor structure for clinical sample

**Fig. 2:**
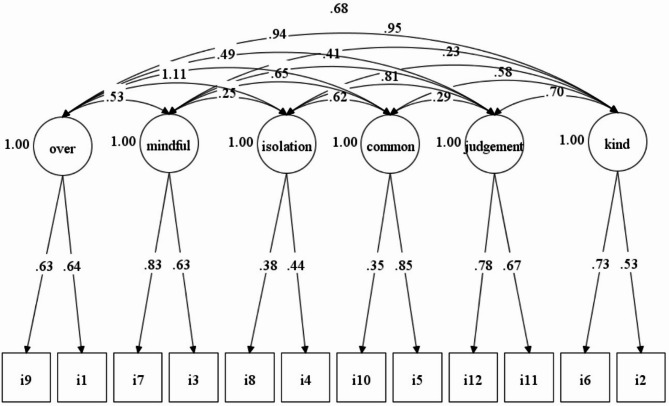
Standardized factor loadings of the items for clinical sample

For the Turkish clinical sample, the best-fitting model was the six-factor model (Table 3). The TLI, CFI, RMSEA, and WRMR values for the six-factor model were 0.968, 0.981, 0.045, and 0.586, respectively. These results demonstrated that the fit indices were better than non-clinical Turkish sample. The standardized factor loadings ranged from 0.35 to 0.85 (Fig. 2).

### Reliability

The Cronbach alpha coefficient of SCS-SF was calculated between 0.77 and 0.88 for the non-clinical, clinical, and American samples of the current study (see Table 1). Using the test-retest reliability method within a two-week interval, SCS-SF scores revealed a strong correlation coefficient (*r* = 0.878, *p* < 0.050). Thus, it was concluded that the SCS-SF scores could be considered reliable over time.

### Convergent validity

To provide the convergent validity evidence of the SCS-SF in the non-clinical sample, the relationships of the SCS-SF scores with both the RSES and the positive relations with others subscales of the SPWB were examined. A marginally strong negative correlation was identified between the SCS-SF and the RSES (*r* = -0.533, 95% BCa CI [-0.616, -0.431], *p* < 0.050; [78]). A medium positive correlation was observed between the SCS-SF and the positive relations with others subscales of the SPWB (*r* = 0.421, 95% BCa CI [0.317, 0.523], *p* < 0.050; [78]). Thus, these correlations were considered as evidence for convergent validity.

To assess the convergent validity of the SCS-SF within a clinical population, the associations between SCS-SF scores and the BDI and the STAI-S were analyzed. It was hypothesized that a negative correlation would exist between the SCS-SF and the BDI and the STAI-S. The analysis revealed a marginally strong negative correlation between the SCS-SF and the BDI (*r* = -0.594, 95% BCa CI [-0.682, -0.495], *p* < 0.050; [78]), as well as between the SCS-SF and the STAI-S (*r* = -0.502, 95% BCa CI [-0.591, -0.401], *p* < 0.050; [78]), thereby supporting the convergent validity.

### Measurement invariance

#### Testing measurement invariance across clinical and non-clinical samples

The measurement model, factor loadings, thresholds, and error variances of the SCS-SF were compared between clinical and non-clinical samples using the measurement invariance test. According to the configural invariance results, the fit indices for TLI = 0.938 and CFI = 0.963, as well as for RMSEA = 0.079 (90% CI = 0.069–0.090), were within the acceptable range. Second, the change in the fit indices supported the metric invariance (ΔCFI = -0.001 < 0.010, ΔRMSEA = 0.004 < 0.015), which was evaluated by restricting factor loadings to be identical. Third, scalar invariance results (ΔCFI = 0.006 < 0.010, ΔRMSEA = 0.005 < 0.015) indicated threshold equality. Lastly, the clinical and non-clinical groups’ error variances appeared to be comparable, according to the strict invariance results (ΔRMSEA = -0.002 < 0.015, ΔCFI = 0.006 < 0.010).

#### Testing measurement invariance across gender groups

For clinical and non-clinical samples, the measurement invariance analysis was conducted to determine whether the measurement model, factor loadings, thresholds, and error variances of the SCS-SF were similar for males and females (see Table 4). For the nonclinical sample, the configural invariance results indicated that the fit indices were below the acceptable value for RMSEA = 0.111 (90% CI = 0.099–0.123), and within an acceptable range for TLI = 0.903 and CFI = 0.943. Second, metric invariance was assessed by constraining factor loadings to be equal and the change in the fit indices supported the metric invariance (ΔCFI = -0.003 < 0.010, ΔRMSEA = 0.007 < 0.015). Third, scalar invariance results supported the equality of thresholds (ΔCFI = 0.002 < 0.010, ΔRMSEA = 0.013 < 0.015). Finally, the strict invariance results suggested that error variances were similar for gender groups (ΔCFI = 0.002 < 0.010, ΔRMSEA = 0.003 < 0.015).

**Table 4:** Multiple group CFA results of the SCS-SF

			X^2^	df	X^2^/df	TLI	CFI	RMSEA (%90 CI)	ΔCFI	ΔRMSEA	Decision
Across clinic vs. non-clinic		Configural	272.312	78	3.491	0.938	0.963	0.079 (0.069–0.090)			
		Metric	272.639	84	3.246	0.944	0.964	0.075 (0.065–0.085)	-0.001	0.004	Accept
		Scalar	334.826	114	2.937	0.952	0.958	0.070 (0.061–0.079)	0.006	0.005	Accept
		Strict	381.701	126	3.029	0.950	0.952	0.072 (0.063–0.080)	0.006	-0.002	Accept
Across the gender (female vs. male)	Non-clinical sample	Configural	338.717	78	4.343	0.903	0.943	0.111 (0.099–0.123)			
		Metric	330.225	84	3.931	0.915	0.946	0.104 (0.092–0.116)	-0.003	0.007	Accept
		Scalar	371.365	114	3.257	0.935	0.944	0.091 (0.081–0.091)	0.002	0.013	Accept
		Strict	391.300	126	3.106	0.939	0.942	0.088 (0.078–0.098)	0.002	0.003	Accept
	Clinical sample	Configural	121.089	78	1.552	0.939	0.964	0.067 (0.042–0.090)			
		Metric	133.702	84	1.591	0.934	0.958	0.069 (0.046–0.091)	0.006	-0.002	Accept
		Scalar	159.681	114	1.400	0.955	0.962	0.057 (0.034–0.077)	-0.004	0.012	Accept
		Strict	197.158	126	1.565	0.937	0.940	0.068 (0.049–0.085)	0.022	-0.011	Reject
Across the culture (Türkiye vs. American) in non-clinical		Configural	265.764	78	3.407	0.941	0.965	0.085 (0.074–0.096)			
		Metric	281.923	84	3.356	0.942	0.963	0.084 (0.073–0.095)	0.002	0.001	Accept
		Scalar	336.263	114	2.949	0.952	0.958	0.076 (0.067–0.086)	0.005	0.008	Accept
		Strict	378.762	126	3.006	0.950	0.953	0.077 (0.069–0.086)	0.005	-0.001	Accept

Note. SCS-SF = Self-Compassion Scale Short Form; TLI = Tucker–Lewis index; CFI = comparative fit index; RMSEA = root mean square error of approximation; CI = confidence interval; ΔCFI = change in values of CFI; ΔRMSEA = change in values of RMSEA

For the clinical sample, the configural invariance results indicated that the fit indices were acceptable for RMSEA = 0.67 (90% CI = 0.042–0.090), for TLI = 0.939, and for CFI = 0.964. The metric invariance results supported the metric invariance (ΔCFI = 0.006 < 0.010, ΔRMSEA = -0.002 < 0.015). Third, scalar invariance results supported the equality of thresholds (ΔCFI = -0.004 < 0.010, ΔRMSEA = 0.012 < 0.015). Finally, the strict invariance results suggested that error variances were not similar for gender groups (ΔCFI = 0.022 < 0.010, ΔRMSEA = -0.011 < 0.015). As a result, for clinical gender groups, the measurement invariance was supported for configural, metric, and scalar, but not for strict invariance.

#### Testing measurement invariance across culture groups

Measurement invariance analysis was conducted to determine whether the measurement model of the SCS-SF was similar for Turkish and American participants. First, configural invariance was assessed and results showed that fit indices were within an acceptable level, TLI = 0.941, CFI = 0.965, RMSEA = 0.085 (90% CI = 0.074–0.096). Second, metric invariance results showed that the change in fit indices was within acceptable range (ΔCFI = 0.002 < 0.010, ΔRMSEA = 0.001 < 0.015). Third, the change in fit indices supported the scalar invariance (ΔCFI = 0.005 < 0.010, ΔRMSEA = 0.008 < 0.015). Finally, strict invariance results suggested that error variances were similar for Turkish and American samples (ΔCFI = 0.005 < 0.010, ΔRMSEA = -0.001 < 0.015).

## Discussion

The current study aimed to adapt the SCS-SF into Turkish and to assess its psychometric properties in clinical and non-clinical samples. The CFA results supported the six-factorial structure in most of the sample. The reliability of the Turkish version of the SCS-SF scale scores were good. Additionally, the results suggested the concurrent validity evidence of the SCS-SF and the measurement invariance of the Turkish version of the SCS-SF was mostly supported across samples of the study (clinical vs. non-clinical, female vs. male, and Turkish vs. American). Strict invariance was not established for only clinical gender groups. Consequently, while the scale is capable of reliably assessing the structure and interrelations of self-compassion across different groups, researchers are advised to exercise caution when interpreting variations in observed scores.

In the current study, one-, two-, six-factor, two-bifactor, and six-bifactor models were tested separately for clinical and non-clinical samples. While only the six-factor model was supported for the clinical sample, the two-bifactor and six-factor models were supported for the non-clinical sample. Thus, the six-factor structure proposed by Raes and colleagues [33] was supported by the clinical and non-clinical Turkish samples of the current study. This finding is consistent with research that found that the six-factor model was supported in English samples [33], Spanish samples [25], and Dutch samples [33]. The factor structure of the SCS-SF appears to be varying between cultures, as suggested by certain research that has found diverse factor structures in various cultural contexts, such as two-factor models found in Canada and Sweden and a three-factor model seen in China. The results of the current study, which used a six-factor model in Türkiye, indicated that different cultures may have different factor structures [29, 79, 80]. Overall, the current results demonstrate that the SCS-SF might serve as a feasible and effective substitute for the SCS in the Turkish context.

The findings of the convergent validity study were found to be moderately related to psychological well-being and self–esteem in the non-clinical sample as hypothesized [81]. In the clinical sample, a moderate negative correlation was found between the BDI and the STAI-S (an important tool for measuring mental health). According to these results, it is revealed that the SCS-SF scores are related to psychological health in non-clinical samples and to psychological disorders in clinical samples in an expected direction. These results are compatible with cross-sectional and experimental studies in the literature [82–84].

### Measurement invariance

The measurement invariance analyses yielded insights regarding the applicability of the Turkish SCS-SF across different demographic groups. As the participant responses provided evidence for metric and scalar invariance for clinical and non-clinical groups, for gender groups (female vs. male), and for cross-cultural samples (Türkiye vs. USA), further studies examining the relationships between the SCS-SF scores or comparing mean scores of self-compassion could be conducted. Nevertheless, strict invariance was not established, only for gender comparisons in clinical sample. These results are consistent with existing research, which sometimes underscores the difficulties in achieving strict invariance in psychological measurement tools. Future enhancements to the scale or supplementary contextual investigations may be necessary to address these discrepancies and improve its applicability across diverse groups.

### Limitations and Recommendation

One of the limitations of the study was its limited sample size for the American sample. The purpose of including American samples in this study was not to compare them cross-culturally but rather to provide additional validity evidence by investigating measurement invariance. As the researchers encountered difficulty finding volunteer participants living in the USA, a small American sample was used. This situation limits the statistical power and generalizability of the findings regarding measurement invariance between the American and Turkish sample. One limitation concerns the potential for subtle semantic drift in abstract psychological constructs, such as *over-identification*, despite rigorous translation procedures. While expert consensus was used to ensure conceptual equivalence, future studies should consider cognitive debriefing interviews or qualitative feedback to evaluate whether respondents interpret such constructs as intended. Another limitation relates to the mode of data collection, as the Turkish data were collected face-to-face while the American data were gathered online. This procedural difference introduces the possibility of mode bias, as participants may respond differently in supervised versus anonymous online settings. Although measurement invariance was tested across cultural groups, we were not able to statistically isolate or control for the influence of mode alone due to sample size constraints in the U.S. group. Future research should aim to collect data using comparable methods across cultures or explicitly test for mode-related effects through multi-group analyses or mode-matched subsamples to better understand the influence of procedural differences on psychometric outcomes. While the inclusion of the U.S. sample offered valuable insights into cross-cultural measurement invariance, the theoretical framing could be expanded in future work by incorporating additional cultural contexts (e.g., East Asian or Latin American) to further explore cultural influences on the factorial validity of the SCS-SF.

## Conclusions

The SCS-SF’s Turkish adaptation demonstrates good psychometric qualities, highlighting its suitability for assessing self-compassion in both clinical and non-clinical Turkish samples. This study is considered to add to the literature by adapting a self-compassion instrument that could provide reliable and valid scores for forthcoming research and practical applications in Türkiye.

The findings of in the current study have several practical implications. For clinicians, the validated Turkish version of the SCS-SF provides a brief and reliable tool to assess self-compassion, which can support the identification of individuals at risk for high self-criticism or low emotional resilience—common in mood and anxiety disorders. The scale may also serve as a monitoring tool in compassion-focused or mindfulness-based interventions. For researchers, the scale offers a culturally adapted instrument suitable for investigating self-compassion in both clinical and non-clinical Turkish populations. Given that measurement invariance was established up to the scalar level, the SCS-SF can be used for latent mean comparisons across gender, clinical status, and cultural contexts, though caution is warranted due to the absence of strict invariance. These results support the scale’s use in cross-cultural and comparative research, while also highlighting the importance of cultural sensitivity in interpreting findings.

The findings support the factorial validity of the Turkish SCS-SF and its general structure across groups, but the lack of strict invariance necessitates caution in using the scale for direct group comparisons. Further refinement or culturally tailored measurement models may be needed to improve score comparability.

The variability in factor structure across cultures emphasizes the need to view self-compassion as a culturally embedded construct. Researchers should be cautious in applying Western-based models without considering cultural adaptation and validation at the conceptual level, not just linguistic translation.

## Electronic supplementary material

Below is the link to the electronic supplementary material.

Supplementary Material 1

Supplementary Material 2

## Data Availability

Büyüköksüz, Engin; Tekin, Işıl; Arıkan, Serkan (2022), “Self-Compassion Scale-Short Form in Clinical and Non-clinical Samples”, Mendeley Data, V1, doi: 10.17632/58my34fsg3.1)
