# Supplementary material for: Psychometric validation and measurement invariance of the self-compassion scale-short form (SCS-SF) across gender, clinical population, and cultures
Source: BMC Psychol. 2025 Jul 1;13:716. doi: 10.1186/s40359-025-03070-8 (PMC12219947; doi:10.1186/s40359-025-03070-8)
Supplement: Supplementary file 1 — Supplementary Material 1 [file 40359_2025_3070_MOESM1_ESM.docx]

**Table 1** Descriptive Information of the Participants

|  | Non-clinical sample Confirmatory Factor Analyses | Clinical sample Confirmatory Factor Analyses | Test-Retest | Correlations with other scales for convergent validity | American non-clinical participant |
| --- | --- | --- | --- | --- | --- |
| n | 545 | 246 | 53 | 274 | 125 |
| Average age (range) | 22.64  (18-45) | 29.51  (18-62) | 20.64  (18-26) | 21.04  (18-43) | 34.60  (18-68) |
| % female | 62.2 %  (n = 339) | 65.9 %  (n = 162) | 79.2 %  (n = 42) | 70.1 %  (n = 192 | 70.4 %  (n = 88) |
| % male | 37.8 %  (n = 206) | 34.1 %  (n = 84) | 20.8 %  (n = 11) | 29.9 %  (n = 82) | 28.0 %  (n = 35) |
| % other | - | - | - | - | 1.6 %  (n = 2) |
| Internal consistency (Cr𝝰) | .81 | .77 |  | .81 | .88 |

**Table 2** Sample items of the SCS-SF

| Subscale | Sample Item in English | Sample Item in Turkish |
| --- | --- | --- |
| Self-Kindness | 2. I try to be understanding and patient towards those aspects of my personality I don’t like. | Kişiliğimin hoşlanmadığım yönlerine karşı anlayışlı ve hoşgörülü olmaya çalışırım. |
| Self-Judgment | 11. I’m disapproving and judgmental about my own flaws and inadequacies. | Sahip olduğum kusurlarımı, yetersizliklerimi onaylamıyorum ve yargılıyorum. |
| Common Humanity | 5. I try to see my failings as part of the human condition. | Başarısızlıklarımı insan doğasının bir parçası olarak görmeye çalışırım. |
| Isolation | 4. When I’m feeling down, I tend to feel like most other people are probably happier than I am. | Kendimi üzgün hissettiğim zaman, çoğu insanın muhtemelen benden daha mutlu olduğunu hissetmeye meyilli olurum |
| Mindfulness | 3. When something painful happens I try to take a balanced view of the situation. | Acı verici bir şey olduğu zaman, durumu dengeleyen bir bakış acısı almaya çalışırım. |
| Over-identified | 1. When I fail at something important to me I become consumed by feelings of inadequacy. | Benim için önemli olan bir şeyde başarısız olduğumda, kendimi yetersizlik duygusuyla tüketirim |

**Table 3** CFA Results of the SCS-SF

| Sample | Models | *X^2^* | *df* | *X^2^/df* | TLI | CFI | RMSEA (%90 CI) | WRMR |
| --- | --- | --- | --- | --- | --- | --- | --- | --- |
| Non-clinical sample | One-factor | 1304.467 | 54 | 24.156 | 0.621 | 0.690 | 0.206 (0.197-0.216) | 3.353 |
|  | Two-factor | 377.003 | 53 | 7.113 | 0.900 | 0.920 | 0.106 (0.096-0.116) | 1.541 |
|  | Six-factor | 230.517 | 39 | 5.910 | 0.920 | 0.953 | 0.095 (0.083-0.107) | 1.115 |
|  | Two-bifactor | 219.242 | 42 | 5.220 | 0.931 | 0.956 | 0.088 (0.077-0.100) | 1.000 |
|  | Six-bifactor | Not identified |  |  |  |  |  |  |
| Clinical sample | One-factor | 263.459 | 54 | 4.882 | 0.753 | 0.798 | 0.13 (0.11-0.14) | 1.364 |
|  | Two-factor | 116.649 | 53 | 2.200 | 0.924 | 0.939 | 0.070 (0.053-0.087) | 0.874 |
|  | Six-factor | 58.611 | 39 | 1.502 | 0.968 | 0.981 | 0.045 (0.017-0.068) | 0.586 |
|  | Two-bifactor | 88.168 | 42 | 2.099 | 0.930 | 0.956 | 0.067 (0.047-0.086) | 0.694 |
|  | Six-bifactor | Not identified |  |  |  |  |  |  |

Note. SCS-SF = Self-Compassion Scale Short Form; TLI = Tucker–Lewis index; CFI = comparative fit index; RMSEA = root mean square error of approximation; CI = confidence interval; WRMR: Weighted Root Mean Square residual

**Table 4** Multiple Group CFA Results of the SCS-SF

|  | |  | *X^2^* | *df* | *X^2^/df* | TLI | CFI | RMSEA (%90 CI) | ΔCFI | ΔRMSEA | Decision |
| --- | --- | --- | --- | --- | --- | --- | --- | --- | --- | --- | --- |
| Across clinic vs non-clinic | | Configural | 272.312 | 78 | 3.491 | 0.938 | 0.963 | 0.079 (0.069-0.090) |  |  |  |
|  |  | Metric | 272.639 | 84 | 3.246 | 0.944 | 0.964 | 0.075 (0.065-0.085) | -0.001 | 0.004 | Accept |
|  |  | Scalar | 334.826 | 114 | 2.937 | 0.952 | 0.958 | 0.070 (0.061-0.079) | 0.006 | 0.005 | Accept |
|  |  | Strict | 381.701 | 126 | 3.029 | 0.950 | 0.952 | 0.072 (0.063-0.080) | 0.006 | -0.002 | Reject |
| Across the gender (female vs male) | Non-clinical sample | Configural | 338.717 | 78 | 4.343 | 0.903 | 0.943 | 0.111 (0.099-0.123) |  |  |  |
|  |  | Metric | 330.225 | 84 | 3.931 | 0.915 | 0.946 | 0.104 (0.092-0.116) | -0.003 | 0.007 | Accept |
|  |  | Scalar | 371.365 | 114 | 3.257 | 0.935 | 0.944 | 0.091 (0.081-0.091) | 0.002 | 0.013 | Accept |
|  |  | Strict | 391.300 | 126 | 3.106 | 0.939 | 0.942 | 0.088 (0.078-0.098) | 0.002 | 0.003 | Reject |
|  | Clinical sample | Configural | 121.089 | 78 | 1.552 | 0.939 | 0.964 | 0.067 (0.042-0.090) |  |  |  |
|  |  | Metric | 133.702 | 84 | 1.591 | 0.934 | 0.958 | 0.069 (0.046-0.091) | 0.006 | -0.002 | Accept |
|  |  | Scalar | 159.681 | 114 | 1.400 | 0.955 | 0.962 | 0.057 (0.034-0.077) | -0.004 | 0.012 | Accept |
|  |  | Strict | 197.158 | 126 | 1.565 | 0.937 | 0.940 | 0.068 (0.049-0.085) | 0.022 | -0.011 | Reject |
| Across the culture (Turkey vs American) in non-clinical | | Configural | 265.764 | 78 | 3.407 | 0.941 | 0.965 | 0.085 (0.074-0.096) |  |  |  |
|  |  | Metric | 281.923 | 84 | 3.356 | 0.942 | 0.963 | 0.084 (0.073-0.095) | 0.002 | 0.001 | Accept |
|  |  | Scalar | 336.263 | 114 | 2.949 | 0.952 | 0.958 | 0.076 (0.067-0.086) | 0.005 | 0.008 | Accept |
|  |  | Strict | 378.762 | 126 | 3.006 | 0.950 | 0.953 | 0.077 (0.069-0.086) | 0.005 | -0.001 | Reject |

Note. SCS-SF = Self-Compassion Scale Short Form; TLI = Tucker–Lewis index; CFI = comparative fit index; RMSEA = root mean square error of approximation; CI = confidence interval; ΔCFI = change in values of CFI; ΔRMSEA = change in values of RMSEA.
