# Supplementary material for: Psychometric validation and measurement invariance of the self-compassion scale-short form (SCS-SF) across gender, clinical population, and cultures
Source: BMC Psychol. 2025 Jul 1;13:716. doi: 10.1186/s40359-025-03070-8 (PMC12219947; doi:10.1186/s40359-025-03070-8)

Figure 1

*Standardized factor loadings of the items for non-clinical sample*


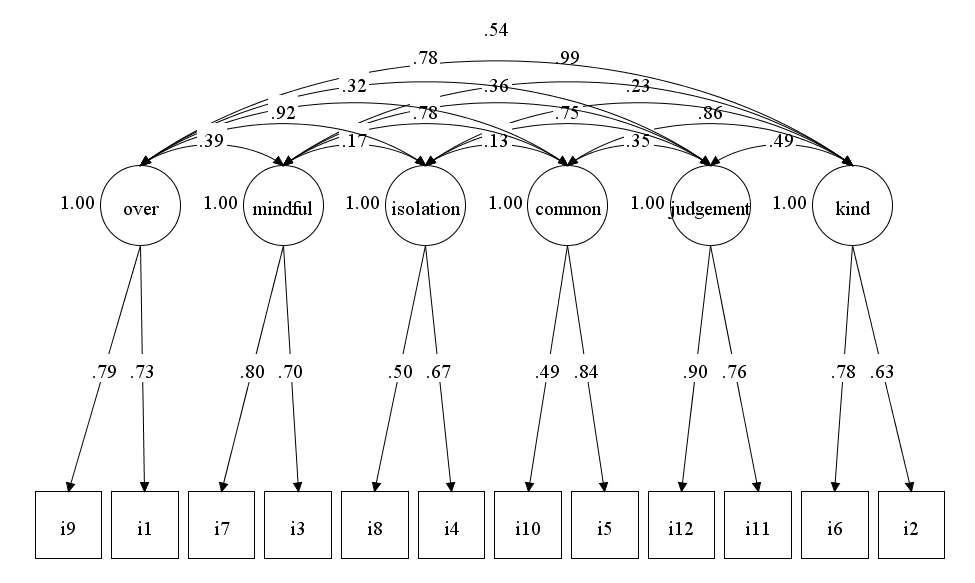


Figure 2

*Standardized factor loadings of the items for clinical sample*


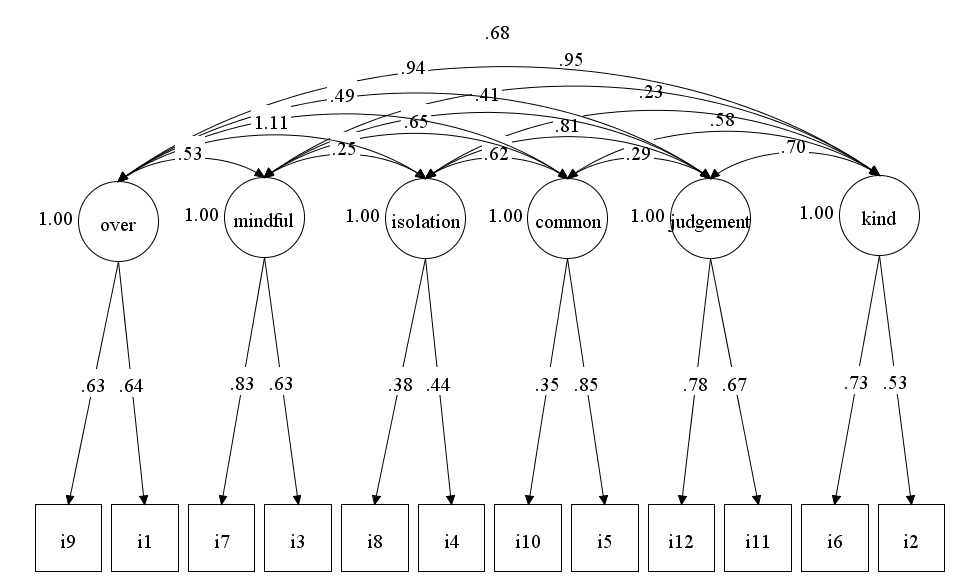

Supplement: Supplementary file 2 — Supplementary Material 2 [file 40359_2025_3070_MOESM2_ESM.docx]
